# Pulse oximetry in the newborn: Is the left hand pre- or post-ductal?

**DOI:** 10.1186/1471-2431-10-35

**Published:** 2010-05-21

**Authors:** Christoph Rüegger, Hans Ulrich Bucher, Romaine Arlettaz Mieth

**Affiliations:** 1 Clinic for Neonatology, University Hospital Zurich, Switzerland

## Abstract

**Background:**

Over the past few years, great efforts have been made to screen duct-dependent congenital heart diseases in the newborn. Arterial pulse oximetry screening (foot and/or right hand) has been put forth as the most useful strategy to prevent circulatory collapse. The left hand, however, has always been ignored, as it was unclear if the ductus arteriosus influences left-hand arterial perfusion. The objective of our study was to evaluate the impact of the arterial duct on neonatal pulse oximetry saturation (POS) on the left hand.

**Methods:**

In this observational study, arterial oxygen saturation on both hands and on one foot was measured within the first 4 hours of life.

**Results:**

Two hundred fifty-one newborns were studied: 53% males and 47% were delivered by caesarean section. The median gestational age was 38 4/7 weeks (90% CI, 32 6/7 - 41 2/7 weeks), the median birth weight was 3140 g (90% CI, 1655 - 4110 g) and the median age at recording was 60 minutes (90% CI, 15 - 210 minutes). The mean POS for the overall study population was 95.7% (90% CI, 90 - 100%) on the right hand, 95.7% (90% CI, 90 - 100%) on the left hand, and 94.9% (90% CI, 86 - 100%) on the foot. Four subgroups (preterm infants, babies with respiratory disorders, neonates delivered by caesarean section, and newborns ≤15 minutes of age) were formed and analysed separately. None of the subgroups showed a statistically significant difference between the right and left hands. Additionally, multivariate logistic regression did not identify any associated factors influencing the POS on the left hand.

**Conclusions:**

With the exception of some children with complex or duct dependent congenital heart defects and some children with persistent pulmonary hypertension, POS on both hands can be considered equally pre-ductal.

## Background

Cardiovascular malformations are the commonest type of congenital malformation (6-8/1000) and responsible for more deaths in the first year of life than any other birth defects. A sixth of the affected children have a duct dependent circulation, with a persistent ductus arteriosus being necessary for survival [1-3]. Over the past few years, great efforts have been made to screen these duct-dependent congenital heart diseases (CHD) in the newborn. As ductal-dependent CHD may not be apparent at the time of early discharge examination [4,5], post-ductal arterial pulse oximetry screening during the first 24 hours of life has been put forth as the most useful strategy to prevent circulatory collapse or death [6-9]. The ease of performing, minimal discomfort for the baby, low costs, and excellent detection rates of duct- dependent circulation are strong arguments to implement pulse oximetry as a basic routine in the nursery [10,11].

In addition, pulse oximetry has been used for observing newborns with respiratory or cardiac disorders and has been found to be helpful in monitoring neonatal resuscitation. In this context, pre-ductal POS, the sensor applied to the right hand, must be used to monitor oxygen application. This is in contrast to the later measured post-ductal POS (foot) to rule out CHD as described above.

For both purposes, previous studies have focused on obtaining pulse oxygen saturation from one foot (post-ductal) and/or the right hand (pre-ductal). The left hand, however, has always been ignored, as it was unclear if the ductus arteriosus influences left-hand arterial perfusion. By means of pulse oximetry measurements, the purpose of the present study was to clarify if values of the left hand can be differentiated from the pre-ductal (right hand) and post-ductal (foot) values.

## Methods

The study population of this observational study included infants born at the University Hospital of Zurich between 5 January and 26 April 2009. Newborn infants with pre- or post-natally diagnosed CHD were also measured, but not included in the overall analysis. The measurements were performed using a Nellcor-65™ handheld pulse oximeter with a neonatal OxiMax adhesive sensor. An accuracy of ±2% for the measurement of functional oxygen saturation (SpO2) was stated by the manufacturer. For each newborn, a neonatologist obtained the POS for both hands and one foot within the first 4 hours of life. The probe was secured to the wrist or palm and to the sole of the foot, following a random order. The same oximeter was used for all three sequential measurements. To avoid movement artifacts, the pulse was observed until a good waveform was obtained. It usually required 3-5 minutes for all 3 measurements to be performed.

As post-ductal (foot) pulse oximetry screening has become the standard of care at our institution, the two additional recordings (right and left hands) were considered a modification of the routine examination. In agreement with the Hospital Committee of Ethics, the parents were informed, but no written consent was obtained. On enrolment in the study, the following information of each infant was recorded: gestational age, gender, birth weight, mode of delivery, umbilical arterial pH, Apgar score at 5 minutes, and postnatal age.

### Statistical analysis

A sample size was estimated based on the number of independent variables included in our multivariate logistic regression. Starting from a minimum number of 10 observations needed for each parameter, a sample size of 110 infants was initially calculated. To be positive about the detection of even small effects, the sample size was doubled to 250. Further, a one-sided paired Student's t test was performed to determine differences between the three measurements, and finally multivariate logistic regression was conducted to identify associated factors. All statistical analyses were carried out with commercial software (Microsoft Excel 2008 for Mac and R, version 2.2.0.2 for Windows).

## Results

During the study period, 739 babies were live born at our institution. Of these newborn infants, 254 (34%) were enrolled in the study. Recordings of the remaining newborns were not obtained for the following reasons: lack of staff, several simultaneous deliveries, and movement artifacts. In addition, three recordings were analysed separately because of known or suspected CHD. The first newborn had trisomy 13 with an interrupted aortic arch; the arterial oxygen saturation showed values of 84%, 79% and 62% on the right hand, left hand, and foot. In a second newborn, echocardiography revealed a double outlet right ventricle with an oxygen saturation of 94%, 88% and 88%, respectively. A third newborn had a saturation of 89%, 86% and 85% on the right hand, left hand, and foot, respectively. A double inlet single ventricle was demonstrated on the echocardiogram.

Consequently, data from 251 infants were recorded and analysed. The median gestational age was 38 4/7 weeks (90% CI, 32 6/7-41 2/7 weeks) and the median birth weight was 3140 g (90% CI, 1655 - 4110 g). Further patient characteristics are given in Table 1. The median age at the beginning of recording was 60 minutes (90% CI, 15-225 minutes).

**Table 1:** Baseline characteristics of the study population.

Characteristics:		Study group:
Female	No. (%)	117 (47)
Male	No. (%)	134 (53)
Gestational Age (week)	p0.5 (p0.05 - p0.95)	38 4/7 (32 6/7 - 41 2/7)
Fullterm	No. (%)	189 (75)
Preterm	No. (%)	62 (25)
Birth weight (g)	p0.5 (p0.05 - p0.95)	3140 (1655 - 4110)
Birth mode		
Spontaneous	No. (%)	98 (39)
Vacuum extraction	No. (%)	34 (14)
Caesarean section	No. (%)	119 (47)
Apgar score at 5 minutes	p0.5 (p0.05 - p0.95)	9 (7 - 9)
Umbilical artery pH	p0.5 (p0.05 - p0.95)	7.3 (7.1 - 7.4)
Age at commencement of recording (min)	p0.5 (p0.05 - p0.95)	60 (15 - 225)

p0.5 = 50th percentile = median, p0.05 = 5th percentile, p0.95 = 95th percentile

Recordings of the study population revealed identical mean oxygen saturations of 95.7% (90% CI, 90 - 100%) on the right and left hands (p-value = 0.41). The post-ductal mean oxygen saturation was 94.9% (90% CI, 86 - 100%), which was 0.8% lower than both hands. In addition, the same analyses were carried out for different subgroups, such as preterm infants, babies with respiratory disorders, neonates delivered by caesarean section, and newborns measured within the first 15 minutes of life. All of the newborn subgroups were at higher risk for an elevated pulmonary artery pressure, and therefore most likely qualified to demonstrate an eventual effect on left-hand perfusion based on right-to-left shunting through the arterial duct. The results of oximetry measurements of the whole study population and the four subgroups are presented in Table 2.

**Table 2:** Arterial oxygen saturation.

Subgroup	Performance	Right hand (%)	Left hand (%)	Foot (%)
All (N = 251)	Mean (90% CI) SD	95.7 (90 - 100) 3.7	95.7 (90 - 100) 4.0	94.9 (86 - 100) 4.6

				

Preterm (N = 62)	Mean (90% CI) SD	94.3 (90 - 100) 5.2	94 (87 - 100) 5.2	93.1 (85 - 100) 5.9

RDS (N = 57)	Mean (90% CI) SD	93.8 (89 - 99) 3.3	93.2 (88 - 98) 4.3	92.6 (85 - 100) 4.8

Caesarean section (N = 119)	Mean (90% CI) SD	95.2 (90 - 100) 4.6	94.9 (88 - 100) 4.8	94.1 (87 - 100) 5.2

Age at recording ≤15 min (N = 34)	Mean (90% CI) SD	95.3 (91 - 100) 3.0	95.1 (90 - 99) 3.2	92.9 (85 - 99) 4.3

				

**Subgroup**	**Performance**	**Right hand -** **Left hand**	**Left hand -** **Foot**	**Right hand -** **Foot**

All	p-value	0.41	<0.001	<0.001
		
Preterm		0.25	<0.001	<0.001
		
RDS		0.06	0.09	0.005
		
Caesarean section		0.12	0.01	<0.001
		
Age at recording ≤15 min		0.37	0.001	<0.001

**Figure 1:**
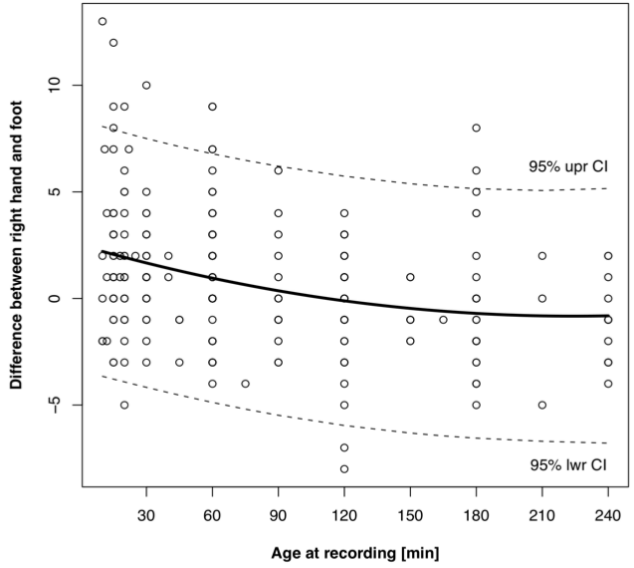
**Difference between right hand and foot correlating with age at recording. **Dotted lines = 5th/95th percentile, solid line = 50th percentile

As described under statistical analysis, the following associated variables for the differences between both hands were identified: Age at recording, respiratory disorder, and caesarean section. None of the variables had a significant impact on the difference between the right and left hand, as shown in Table 3 (p-values > 0.05). For the differences between the right hand and the foot, birth weight and age at recording remained in the model [Table 4]. These differences were shown to decreases with postnatal age, as plotted in Figure 1. Again, the caesarean section was not suitable for further analysis.

**Table 3:** Effects of variables on the POS difference between the right- and the left hand.

Variable:	numDF	denDF	F-value	p-value
Age at recording	1	56	3.374	0.058
Respiratory disorder	1	56	3.355	0.065
Mode of delivery (caesarean section)	2	56	3.181	0.174

numDF = degrees of freedom numerator, denDF = degrees of freedom denominator

**Table 4:** Effects of variables on the POS difference between the right hand and the foot.

Variable:	numDF	denDF	F-value	p-value
Age at recording	1	55	5.016	0.029
Birth weight	1	55	7.046	0.010
Mode of delivery (caesarean section)	2	55	0.734	0.484

numDF = degrees of freedom numerator, denDF = degrees of freedom denominator

## Discussion

### Right Hand - Left Hand

The present study demonstrates that arterial pulse oximetry measurements on the left hand do not significantly differ from the pre-ductal values on the right hand. Even with regard to the four subgroups, in which right-to-left shunting through fetal circulatory pathways resulting from persistent pulmonary hypertension can be assumed, no statistically significant difference was found. We conclude from our data that perfusion of the left hand is unaffected by the arterial duct and can be considered pre-ductal.

In the subgroup consisting of babies with respiratory disorders, a trend, but no statistically significant difference between both hands was detected (p-value 0.06). This trend towards lower values on the left hand can physiologically be explained by a delayed decrease in pulmonary hypertension.

### Right Hand - Foot

Other results fully correspond to those of previous studies. Our overall post-ductal POS levels were 0.8% lower compared to the pre-ductal POS levels [12,13]. In fact, the subgroup consisting of babies recorded within the first 15 minutes of life had post-ductal values that were 2.4% lower. An elevated pulmonary artery pressure during the first minutes of life is the main cause for this difference. Levesque et al [12] reported that postnatal age and the activity state, for example fussy and crying infants at the time of measurement were the most important factors affecting post-ductal POS. In the present study, multivariate logistic regression detected age at recording as one of two variables influencing the difference between pre- and post-ductal values. This is consistent with findings of other studies, in which POS has been shown to increase with time [13-15]. The second variable derived from multivariate logistic regression was birth weight, with a positive correlation to the difference between pre- and post-ductal values. This was unexpected and could neither be explained conclusively nor brought in line with the results of previous studies.

Other authors have focused on whether or not the mode of delivery could have an effect on saturation [14-16]. They all postulated lower post-ductal saturations due to greater retained fetal lung fluid after caesarean section. This is confirmed by our data which showed a significantly lower mean post-ductal saturation in infants delivered by caesarean section (94.1%) compared to infants delivered vaginally (96%, p-value = 0.002). The difference between pre- and post-ductal values, however, was not significantly influenced by the mode of delivery.

## Conclusions

Arterial pulse oximetry is a powerful tool to screen for life threatening CHD. Within the wide range of pre- and post-ductal oxygen saturation, the present study clearly demonstrates that even during the first hours of life, characterised by the process of adaptation, the right and left hand do not significantly differ. We therefore conclude that with the exception of some children with complex or duct dependent CHD and some children with persistent pulmonary hypertension, POS on both hands can be considered equally pre-ductal.

## Competing interests

The authors declare that they have no competing interests.

## Authors' contributions

CR collected and analysed the data, and wrote the manuscript. HUB performed the statistical analysis and participated with RAM in the conception of the study. Both HUB and RAM helped to interpret the data. All the authors read and approved the final manuscript.

## Pre-publication history

The pre-publication history for this paper can be accessed here:

http://www.biomedcentral.com/1471-2431/10/35/prepub
